# Fast, Precise, and Reliable Multiplex Detection of Potato Viruses by Loop-Mediated Isothermal Amplification

**DOI:** 10.3390/ijms21228741

**Published:** 2020-11-19

**Authors:** Güven Edgü, Lena Julie Freund, Stefanie Hartje, Eckhard Tacke, Hans-Reinhard Hofferbert, Richard M. Twyman, Gundula A. Noll, Jost Muth, Dirk Prüfer

**Affiliations:** 1 Fraunhofer Institute for Molecular Biology and Applied Ecology IME, Forckenbeckstrasse 6, 52074 Aachen, Germany; Gueven.Edgue@ime.fraunhofer.de (G.E.); Lena.Julie.Freund@ime.fraunhofer.de (L.J.F.); jost.muth@ime.fraunhofer.de (J.M.); 2 Böhm-Nordkartoffel Agrarproduktion GmbH&Co. OHG, Brüggerfeld 44, 29574 Ebstorf, Germany; shartje@bna-kartoffel.de (S.H.); etacke@bna-kartoffel.de (E.T.); hrhofferbert@bna-kartoffel.de (H.-R.H.); 3 Twyman Research Management Ltd., P.O. Box 493, Scarborough YO11 9FJ, UK; richard@twymanrm.com; 4 Institute of Plant Biology and Biotechnology, University of Münster, Schlossplatz 8, 48143 Münster, Germany; gnoll@uni-muenster.de

**Keywords:** loop-mediated isothermal amplification, lateral-flow dipstick, tobacco rattle virus

## Abstract

Potato is an important staple food crop in both developed and developing countries. However, potato plants are susceptible to several economically important viruses that reduce yields by up to 50% and affect tuber quality. One of the major threats is corky ringspot, which is a tuber necrosis caused by tobacco rattle virus (TRV). The appearance of corky ringspot symptoms on tubers prior to commercialization results in ≈ 45% of the tubers being downgraded in quality and value, while ≈ 55% are declared unsaleable. To improve current disease management practices, we have developed simple diagnostic methods for the reliable detection of TRV without RNA purification, involving minimalized sample handling (mini), subsequent improved colorimetric loop-mediated isothermal amplification (LAMP), and final verification by lateral-flow dipstick (LFD) analysis. Having optimized the mini-LAMP-LFD approach for the sensitive and specific detection of TRV, we confirmed the reliability and robustness of this approach by the simultaneous detection of TRV and other harmful viruses in duplex LAMP reactions. Therefore, our new approach offers breeders, producers, and farmers an inexpensive and efficient new platform for disease management in potato breeding and cultivation.

## 1. Introduction

Potato (*Solanum tuberosum*) is an annual flowering plant belonging to the nightshade family (Solanaceae), which comprises ≈100 genera and ≈2700 species including tomato and tobacco [1]. Its starch-rich tubers were cultivated in the Andes for ≈ 8000 years before their introduction to the rest of the world in the 16th century by European explorers [2] (pp. 11–14). Potato is now farmed on ≈17.5 million ha worldwide with an annual production volume of 350–375 million tons [3], making it the most important staple food after maize, wheat, and rice [4]. Potato prices are usually determined by regional production costs, and (unlike the major cereals) negligible quantities are traded internationally [2] (p. 128). Accordingly, the Food and Agriculture Organization (FAO) recommends potato as a security crop that could be used to support the growing world population and the associated food demand and supply issues, especially in low and middle-income settings [4]. Potato is a valuable cash crop for millions of farmers because it is easy to cultivate and has a high energy content, with developing countries now accounting for more than half of the global output [2] (p. 3).

Corky ringspot is a serious vector-borne potato disease caused by tobacco rattle virus (TRV), which is spread by nematodes [5]. TRV belongs to the genus *Tobravirus* and its genome comprises two single-stranded RNA molecules: RNA1 (6791 nt) and RNA2 (3685 nt) [6]. The non-multiplying RNA1 (NM-type) encoding the 29 kDa movement protein is sufficient for infection and the appearance of symptoms [7], whereas RNA2 encoding the coat protein is not required for systemic propagation [8]. Local necrotic lesions in the tuber manifest as rust-like concentric patterns and/or mottling spots. The appearance of such lesions before commercialization causes ≈45% of tubers to be downgraded in quality and value while ≈55% are declared unmarketable [4,9,10]. Efficient nematicides against the TRV vector are unavailable to most farmers or economically inaccessible due to the high treatment costs of €525/ha [11]. TRV has a broad host range, with more than 100 species susceptible naturally and more than 400 under laboratory conditions, thus exacerbating the challenge of disease prevention and management [8,12].

TRV antisera are commercially available, but immunological detection methods such as the enzyme-linked immunosorbent assay (ELISA) are inefficient and inaccurate because the coat protein is absent from NM-type infections. Therefore, RT-PCR remains the gold standard for TRV detection [13] despite the laborious nature of RNA extraction, reverse transcription, cDNA amplification by PCR, and final product verification by gel electrophoresis. Furthermore, the small samples used for PCR-based diagnostics present a risk of false-negative results if the virus is not homogeneously distributed in the tuber tissue. Finally, samples must be sent to a central laboratory for processing due to the requirement for state-of-the-art thermocyclers, whereas on-site diagnostic methods would be much more convenient. These limitations have been addressed by the development of isothermal amplification methods that achieve rapid detection without complex and expensive equipment [14].

**Figure 1:**
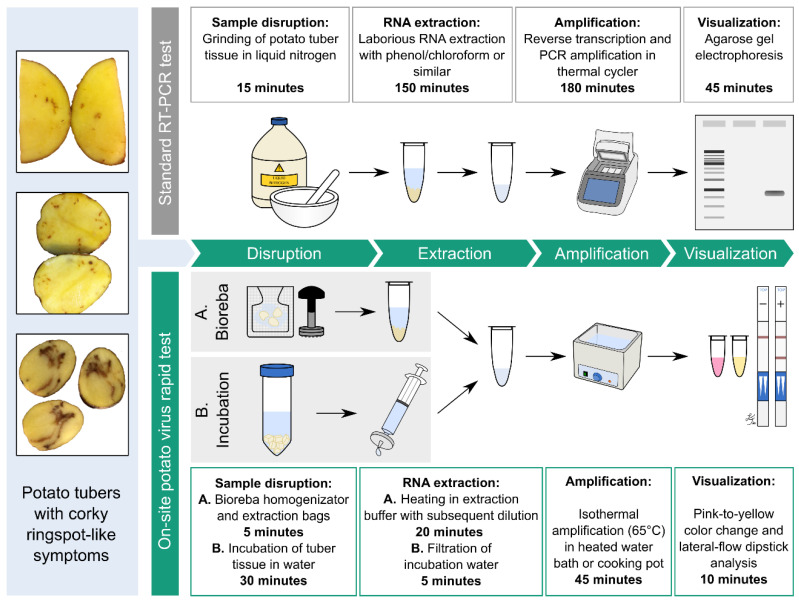
Comparison of the mini-loop-mediated isothermal amplification (LAMP)-lateral-flow dipstick (LFD) approach and standard qRT-PCR. The mini-LAMP-LFD rapid test uses reverse transcription loop-mediated isothermal amplification (RT-LAMP), which in contrast to qRT-PCR does not require purified RNA (minimalized sample handling = mini) and is carried out at a constant temperature (60–65 °C) without expensive laboratory equipment. The use of labeled LAMP detection probes allows final amplification products to be verified by lateral-flow dipstick (LFD) analysis (similar to a pregnancy test) to distinguish between true and false positives.

In this study, we tested several commercial kits for isothermal amplification as alternative nucleic acid amplification tests (NAATs) for the potential on-site detection of agricultural pathogens such as TRV, focusing on the principles of recombinase–polymerase amplification (RPA) [15], thermophilic helicase-dependent amplification (tHDA) [16], CRISPR/Cas9 nickase strand displacement amplification (CRISDA) [17], and loop-mediated amplification (LAMP) [18]. As templates, we used purified viral RNA or tuber/leaf extracts derived from reference material provided by the DSMZ (German Collection of Microorganisms and Cell Cultures) at the Leibniz Institute (Braunschweig, Germany). Comparative evaluation revealed that LAMP was superior in terms of specificity, sensitivity, and its ability to tolerate the presence of PCR/NAAT inhibitors. Therefore, we compared multiple LAMP kits and optimized the sample preparation and processing steps. We compared different primer sets, target/template buffers, reaction supplements (betaine, dimethyl sulfoxide (DMSO), guanidine hydrochloride, Q5 polymerase, and *Tte* UvrD helicase) and low-effort sampling methods, resulting in the development of standard operation procedures for two simple, convenient, and inexpensive on-site potato virus tests (Figure 1). Accordingly, we were able to close the diagnostic gap for the detection of asymptomatic systemic potato infections, enabling the rapid and sensitive detection of all TRV types without expensive equipment, and the simultaneous identification of other pathogens such as potato virus X (PVX). Our results will help to distinguish virus-induced corky ringspot from physiological damage and thus help to enable phytosanitary measures such as weed control, cultivation of resistant varieties, and broad crop rotation in a timely manner [19], as well as maintaining the health of potato varieties throughout the entire propagation and cultivation chain, minimizing the risk of undetected diseases.

## 2. Results

### 2.1. Prioritization of Loop-Mediated Isothermal Amplification (LAMP) as an Alternative Nucleic Acid Amplification Test (NAAT) for Potato Virus Detection

**Figure 2:**
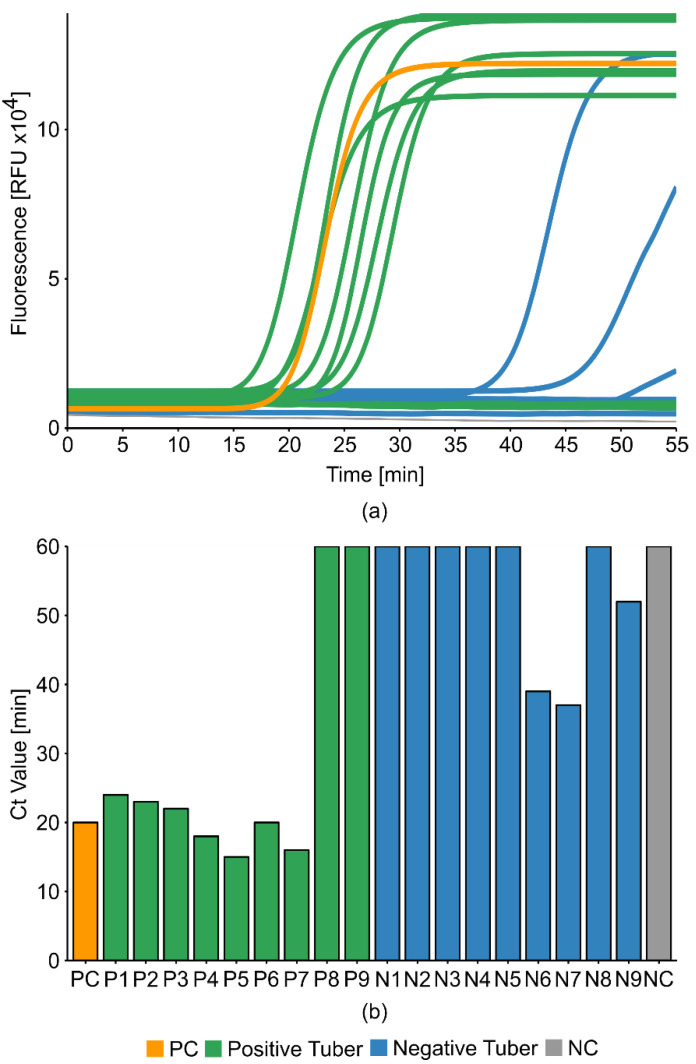
Initial testing of the tobacco rattle virus (TRV)-LAMP method. The NEB WarmStart qRT-LAMP method was tested with HPLC-purified primer mix 1 (PM1). Template = RNA purified from TRV-infected (positive, green) and uninfected (negative, blue) tuber tissues. The total reaction volume was 10 µL (including 0.5 µL template, 0.5 µL primer mix and 0.1 µL 50× LAMP dye). Positive control (PC, orange) = German Collection of Microorganisms and Cell Cultures (DSMZ) virus isolate TRV PV-0352. Negative non-template control (NC, gray) = milliQ water. For visual clarity, the amplification plots (**a**) show the increase in fluorescence with the number of cycles, and the bar chart (**b**) shows the overall Ct values. RT-LAMP tests were monitored for 1 h (1 min/cycle).

Total RNA was extracted and purified from nine healthy and nine naturally TRV-infected tubers obtained from field-grown potato plants using PureLink Plant RNA Reagent (Thermo Fisher Scientific, Waltham, MA, USA) according to the manufacturer’s instructions, which is hereafter described as Extraction Method 1. To evaluate the performance of different isothermal detection methods compared to RT-PCR, we prepared side-by-side reactions based on the principles of tHDA, RPA, CRISDA, and LAMP. The results were monitored by real-time amplification and visualized by agarose gel electrophoresis. The tHDA, CRISDA, and TwistDx-RPA kits did not generate any amplicons, whereas the Agdia-RPA kit generated weak and inconsistent results (data not shown). In contrast, the LAMP method (WarmStart LAMP 2× Master Mix from New England Biolabs (NEB), Ipswich, MA, USA) achieved promising initial results, correctly detecting seven of the nine infected tubers. The WarmStart LAMP method also generated three false-positive by-products, but these clearly differed from genuine amplicons in terms of cycle threshold (Ct) values, with delays exceeding 10, 20, and 25 min, respectively (Figure 2).

### 2.2. Iterative LAMP Optimization for the Improved Detection of Potato Viruses

#### 2.2.1. Comparison of Commercial LAMP Kits and Final Selection for Assay Development

Given the superior performance of the LAMP method in the initial experiments with purified RNA samples, we next compared a range of commercially available LAMP kits. Isolated nucleic acids, minimally processed tuber and leaf material, and extracts of infected plant material were used as templates. We found that the WarmStart Master Mix (NEB) was superior to the GspSSD2.0 Isothermal Mastermix (OptiGene, Horsham, UK) and LavaLAMP Kit (LGC, Teddington, UK) but was partially outperformed by the colorimetric LAMP system also provided by NEB. Therefore, all subsequent experiments and optimization steps were initially carried out using the NEB WarmStart LAMP kit and ultimately the colorimetric WarmStart LAMP kit. 

#### 2.2.2. Comparison and Optimization of LAMP Primer Sets

**Figure 3:**
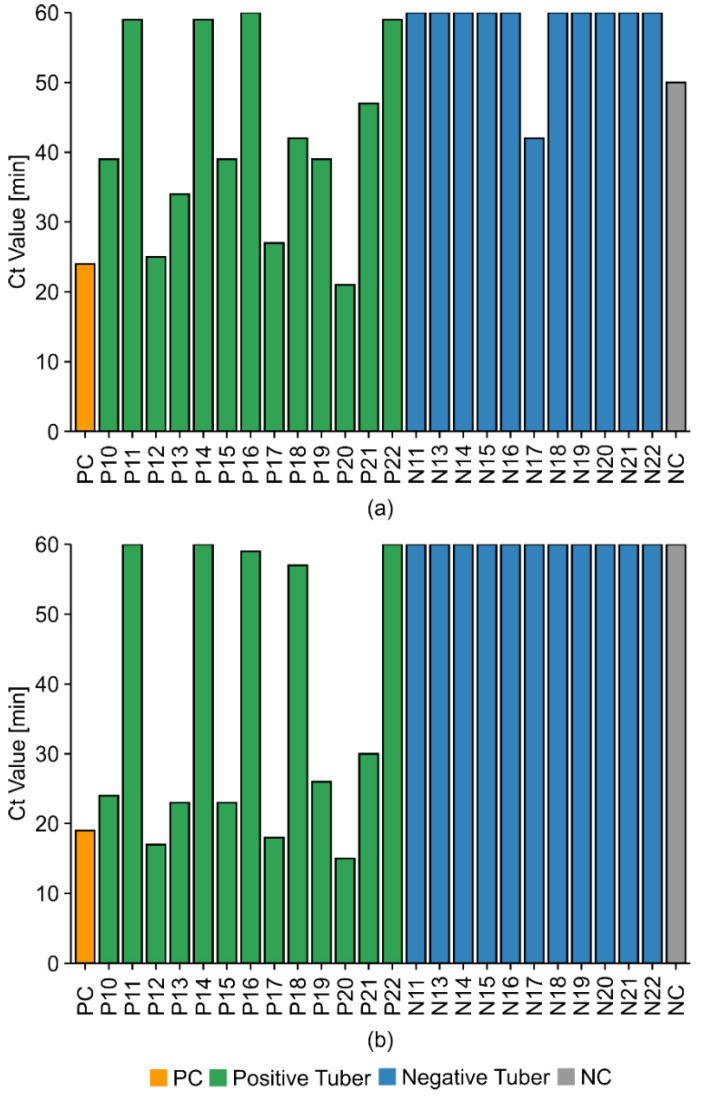
TRV LAMP primer mix comparison. NEB WarmStart qRT-LAMP tests with (**a**) HPLC-purified primer mix 1 (TRV-PM1) or (**b**) TRV-PM2 with new forward internal primer (FIP) and backward internal primer (BIP). Template = RNA purified from TRV-infected (positive, green) and uninfected (negative, blue) tuber tissues. We used 1 µL (1:10 dilution) of template together with positive and negative controls in a total volume of 10 µL. Positive control (PC, orange) = DSMZ virus isolate TRV PV-0352. Negative non-template control (NC, gray) = milliQ water. RT-LAMP tests were monitored for 1 h (1 min/cycle).

We initially compared six TRV-LAMP primer sets designed using Primer Explorer [20] to match the TRV-RNA1 region encoding the movement protein, but these performed poorly. Therefore, we designed primer mix 1 (TRV-PM1) according to the LAMP primer design guidelines provided by Lucigen (Middleton, WI, USA) [21]. TRV-PM1 was successful but provided room for improvement because three false positives were generated from healthy tuber samples (Figure 2). Therefore, we replaced the mixed-nucleotide positions in TRV-PM1 (representing natural sequence variations among virus isolates) with the natural base deoxyinosine, which pairs with all four standard DNA bases [22] (see Table S1, Figure S1). Then, we modified the TRV-PM1 forward internal primer (FIP) and backward internal primer (BIP) by replacing the F2 and B2 binding regions with more promising target sequences that were identified in experiments investigating further TRV primer sets (data not shown). This new oligonucleotide mix (TRV-PM2) was tested against TRV-PM1 using template RNA purified from 13 positive and 11 negative tubers (determined by RT-PCR) in WarmStart LAMP reactions (Figure 3).

TRV-PM1 correctly detected 9/13 TRV-positive tubers with a mean cycle threshold (Ct) of ≈ 39 min but also generated two false-positive products. TRV-PM2, differing from TRV-PM1 only in the F2 and B2 regions, correctly detected 8/13 TRV-positive samples but did not generate any false positives while achieving the fastest mean Ct of 25 min. In subsequent experiments with ambiguous samples, we investigated whether replacing the F3 and/or B3 primers might improve the performance even further. One of these compositions (TRV-PM3), differing from TRV-PM2 only in the B3 component, slightly improved the mean Ct by ≈ 2 min while generating no false-positive results (data not shown). Therefore, TRV-PM3 was used for all subsequent experiments.

#### 2.2.3. General Optimization of LAMP Reactions

Unless specified otherwise, all LAMP reactions discussed herein used templates prepared in milliQ water to ensure comparability. However, reagents such as betaine [23,24] and DMSO [25] or HPLC purification of at least FIP and BIP primers [26] can improve the performance of NAATs [27], so we tested these additional components in our LAMP reactions. We used the PVX LAMP assay to highlight the importance of additives and high-quality LAMP probes by comparing PVX primer mix 4 (PVX-PM4) prepared by standard desalting against new HPLC-purified counterparts. Table 1 not only summarizes the effect of betaine and DMSO on the performance of the LAMP reactions but further demonstrates the unexpected finding that HPLC purification rather than standard desalting of the LAMP oligonucleotides noteworthy improves the reaction specificity and speed compared to standard desalting.

We found that the mean detection time of four different PVX isolates, each tested at two dilutions (1/200 and 1/1600), was successfully reduced from ≈28 min (for the routine desalted primers) to ≈16 min when using the best-performing HPLC-purified primers also including loop primers (full PVX-PM4) in combination with 450 mM betaine (but not 7.5% DMSO). DSMZ inoculum PV-0014 dilutions achieved the most impressive results because the replacement of standard PVX-PM4 with HPLC-purified counterparts initially decreased the Ct by 5 min. The further inclusion of 0.4 µM 4.PVX-FL and 4.PVX-BL halved the Ct from 29 to <16 min. 

We used TRV-PM3 (without loop primers) and final PVX-PM4 (including FL/BL primers) for further LAMP optimization. The addition of as little as 0.15 U of high-fidelity DNA polymerase to a standard 25 µL LAMP reaction mixture was recently shown to increase sensitivity for four Dengue serotypes by removing mismatched bases at the 3′-end of the primers [28]. Furthermore, *Tte* UvrD helicase (NEB) was predicted to improve the specificity of isothermal amplifications, particularly when using WarmStart LAMP kits [29,30]. Therefore, we tested TRV-LAMP reactions with and without the recommended quantities of Q5 polymerase or helicase in our optimized LAMP protocol. No significant improvements were observed, and indeed, both additives resulted in specific drawbacks, so we excluded them from subsequent experiments (Figure S2). The inclusion of Q5 generated new false positives, whereas the inclusion of helicase not only reduced non-specific product formation but also the amplification of specific products, highlighting the caution required when working with plant tissues and enzymatic supplements.

Finally, we tested the addition of deoxyuridine triphosphate (dUTP) and uracil DNA glycosylase (UDG) to prevent carry-over contamination using recommended UTP/UDG concentrations [31,32]. We found that these additives had no effect on reaction speed or sensitivity but increased the specificity (fewer false positives), so we therefore incorporated the UTP/UDG system into subsequent experiments (Figure S3). 

#### 2.2.4. The Sensitivity of the Optimized LAMP Reaction Protocol

To determine the limit of detection (LOD) of the TRV-LAMP method, RNA was purified from a severely infected tuber sample (P10, confirmed by RT-PCR) using Extraction Method 1. Initially, two-fold serial dilutions of the P10 template (ranging from 10 ng/µL to 5 pg/µL RNA) were prepared and analyzed, using a target input of 20% in a final reaction volume of 10 µL.

**Figure 4:**
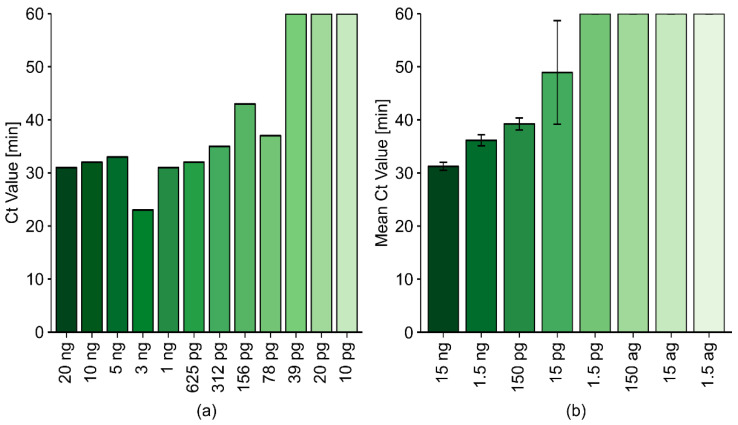
LAMP optimization to determine the limit of detection (LOD). NEB WarmStart qRT-LAMP tests were carried out using (**a**) TRV-PM3 or (**b**) TRV-PM3 plus loop primers. Betaine was added to a final concentration of 800 mM. Template = RNA purified from sample P10 (TRV-positive potato tuber), 1:2 (**a**) or 1:10 (**b**) dilution series. The reaction volume was 10 µL, including 2 µL of the template. Data are mean Ct values plus standard deviations (*n* = 3).

Using our iteratively improved protocol based on HPLC-purified TRV-PM3 with 800 mM betaine in the reaction mix, we achieved an LOD of 78 pg, which is similar to the sensitivity of RT-PCR (Figure 4a). Based on the results achieved for PVX (Table 1), we designed TRV-specific forward loop (FL) and backward loop (BL) primers for TRV-PM3 and tested the new mix in a second LOD experiment (Figure 4b). Under identical reaction conditions (other than the inclusion of the new loop primers, 0.4 µM each), the LOD fell to ≈15 pg. Despite large differences in template concentration, the LAMP assays were completed within 1 h and produced a near-constant final fluorescence intensity, even for low copy number samples, thus achieving the more reliable visualization of positive results compared to agarose gels (where the presence of weak bands may indicate true positives or spillover from adjacent lanes). Therefore, classical gel-based visualization was discontinued for subsequent experiments, except where necessary for the verification of LAMP results based on modified methods. Finally, we used this optimized RT-LAMP protocol to test 118 RNA samples (89 positive and 29 negative, as confirmed by RT-PCR). We detected 79 of the 89 positive samples, and all of the negative samples were correctly identified as such, resulting in a final sensitivity of 89% and a final specificity of 100%.

### 2.3. Lateral-Flow Dipsticks for Post-LAMP Result Verification

**Figure 5:**
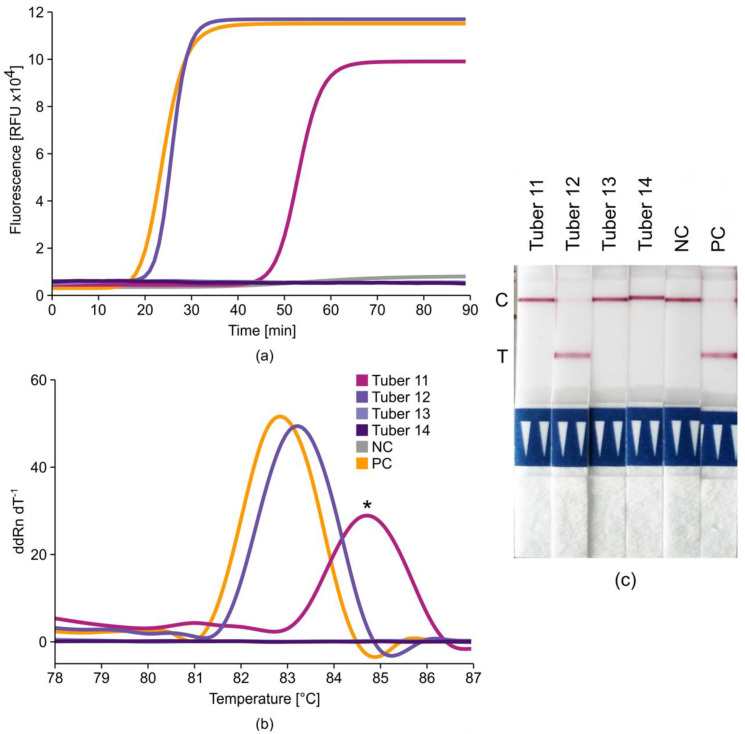
Specificity of RT-LAMP determined by melt curve analysis and lateral-flow dipsticks. RNA purified from four samples (Tuber 11–14) was analyzed by RT-LAMP with TRV-PM3 plus loop primers including 5′ fluorescein amidite (FAM)-labeled FL and 5′ biotin-labeled FIP. DSMZ virus isolate PV-0352 was used as positive control (PC, orange) and MilliQ water was used as non-template negative control (NC, gray). (**a**) Real-time fluorescence measurement of amplification. RFU = raw fluorescence unit. (**b**) Melt curve analysis of amplification products (ddRn dT^−1^ = first derivative of normalized fluorescence intensity). * Nonspecific melting temperature. (**c**) Lateral-flow dipstick with internal dipstick control line (top line) and test line (bottom line, TRV positive). C = internal dipstick control line, T = TRV positive test line.

The specificity of LAMP detection was initially confirmed by melt curve analysis [33], thus helping to establish our mini-LAMP protocol. We also tested other verification methods on ambiguous LAMP products. For example, restriction digests efficiently distinguished true and false positives where appropriate restriction sites were available in the amplicon, although this would be expensive and time-consuming for routine analysis, adding at least 1 h to each experiment (data not shown). In medical diagnostics, lateral-flow dipstick (LFD) technology is widely used in point-of-care devices such as pregnancy tests, providing results in minutes [34]. The LFD method can specifically detect and visualize nucleic acid targets [35], so we modified TRV-PM3 (plus loop primers) to include 5′ fluorescein amidite (FAM)-labeled forward loop (FL) and 5′ biotin-labeled FIP primers in place of the unlabeled originals. RNA samples from one severely infected and thus unambiguously positive sample and three further samples that had initially provided intriguing false-positive signals (very high Ct values in qRT-PCR and qRT-LAMP experiments, data not shown) were randomized, assigned names (Tuber 11–14), and analyzed by qRT-LAMP using TRV-PM3 plus loop primers and 800 mM betaine (Figure 5a). Subsequent melt curve analysis (Figure 5b) was followed by LFD verification (Figure 5c).

Among the three ambiguous samples, Tuber 11 was the only one to generate a spurious amplification product, leading to a late but measurable amplification (high Ct compared to Tuber 12 and the positive control), clearly indicating nonspecific product formation (Figure 5a). Melt curve analysis (Figure 5b) revealed a 2 °C difference between the specific amplification products of TRV-positive Tuber 12 and the positive control (≈83 °C, Ct ≈ 23 min in both cases) compared to the ambiguous sample Tuber 11 (≈85 °C, Ct ≈ 52 min), again suggesting a nonspecific product because these usually show different melting temperatures [33,36]. We intentionally equipped the TRV-PM3 FIP and FL primers with different labels because only amplification products containing both labeled primers can be detected using our LFD method. Therefore, nonspecific or off-target by-products are unmasked as such without needing a qPCR device capable of melt curve analysis. Furthermore, the LFD method is superior to melt curve analysis because interfering extract components or differences in salt concentration and evaporation that lead to variations in Tm (or nonspecific amplification products with similar Tm) do not influence the dipstick verification of LAMP products.

### 2.4. Tobacco Rattle Virus (TRV)+ Potato Virus X (PVX) Duplex LAMP Assays

**Figure 6:**
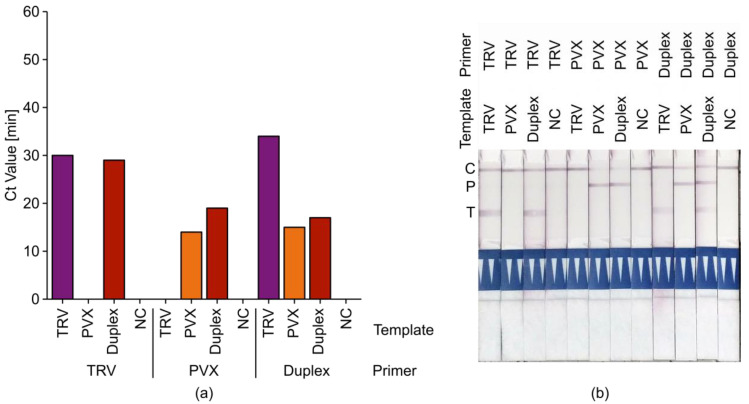
Duplex RT-LAMP assay for TRV and PVX. (**a**) Ct values for the qRT-LAMP analysis of DSMZ virus isolates PV-0352 (TRV, purple), PV-0014 (PVX, orange), or both (duplex, red) with FAM/biotin-labeled TRV-PM3 (TRV), FAM/digoxigenin (DIG)-labeled PVX-PM4 (PVX) or both (duplex). MilliQ water was used as non-template negative control (NC). One cycle = 1 min. (**b**) Lateral-flow dipstick analysis of amplification products with two test lines: T (bottom line) = TRV positive, P (middle line) = PVX positive, and C (upper line) = internal dipstick control.

Next, we assessed the potential of duplex LAMP reactions that can detect more than one pathogen in a single assay. As a proof of concept, we combined primers for the detection of PVX and TRV using virus isolate mixtures as templates. Initial experiments showed that PVX amplicons were produced more efficiently (Figure S4), so we adjusted the primer concentrations accordingly to achieve a better balance. As shown in Figure 6, the specific amplification of TRV and PVX products was possible on mixed templates without cross-reactivity or nonspecific amplification. Dipsticks with two different test lines allowed the specific detection of both amplification products. 

### 2.5. Introduction of Minimal Sampling Methods for the Potential On-Site Screening of Potato Viruses

Having established a reliable TRV-LAMP protocol with comparable or even superior efficiency to the RT-PCR gold standard when applied to purified RNA samples, we investigated the possibility of simplifying the standard RNA preparation method. As discussed above, this standard method (Extraction Method 1) requires liquid nitrogen for sample preparation and uses PureLink Plant RNA Reagent. A streamlined method involving minimally processed tuber and/or leaf tissue could allow screening to be carried out in the field using simple equipment without the need for highly skilled personnel. 

#### Rapid Extraction of Potato Nucleic Acids Using Bioreba Components

**Figure 7:**
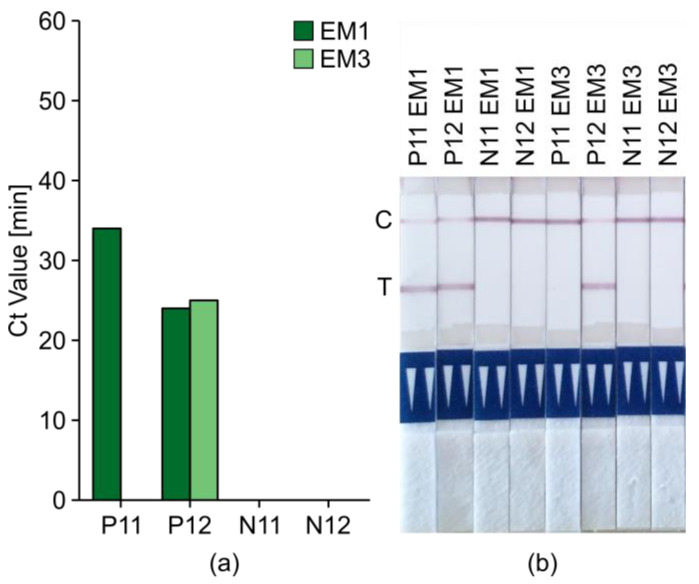
Comparison of standard and Bioreba extraction methods. (**a**) Ct values for qRT-LAMP assays with FAM/biotin-labeled PM3. The template RNA was purified using the standard method (Extraction Method 1) based on the PureLink Plant RNA Reagent (EM1, dark green) or Extraction Method 3 based on the Bioreba rapid extraction kit (EM3, light green). Four samples were tested: two TRV-positive tubers (P11 and P12) and two negative tubers (N11 and N12). One cycle = 1 min. (**b**) Lateral-flow dipstick analysis with internal dipstick control line (C, upper line) and test line (T, lower line, TRV positive).

Bioreba (Reinach, Switzerland) provides extraction bags and a handheld homogenizer that can be used to extract RNA from potato tissues without the use of liquid nitrogen, as well as a rapid extraction kit for potato nucleic acids designed for diagnostic use and validated according to ISO/IEC 17025. We evaluated one method (Extraction Method 2) in which the extraction bags were combined with the PureLink Plant RNA Reagent protocol from Extraction Method 1. We also evaluated the rapid extraction kit, in which extraction from potato tissue is achieved in five steps using two buffers, a homogenizer, and extraction bags (Extraction Method 3). Then, we compared Extraction Methods 1 and 3 side-by-side in tests on four different potato tubers (two healthy, one heavily infected, and one with ambiguous false-positive RT-LAMP results) using HPLC-purified PM3 plus loop primers and 800 mM betaine with 2 µL of template (either ≈ 40 ng of total RNA or the Bioreba extract) followed by direct LFD analysis (Figure 7).

Extraction Method 3 was able to detect positive sample P12 with a slight but negligible amplification delay compared to Extraction Method 1, and no false positives were apparent. P11 (which generated ambiguous initial results, Table S2) tested positive when using Extraction Method 1 but negative when using Extraction Method 3. All LAMP results were verified by subsequent LFD analysis. To conclude, Extraction Method 3 was fast and reliable (no false positives) for sample preparation prior to LAMP tests and was therefore selected for subsequent experiments. 

### 2.6. Potato Tuber Incubation Samples (InCus)

**Figure 8:**
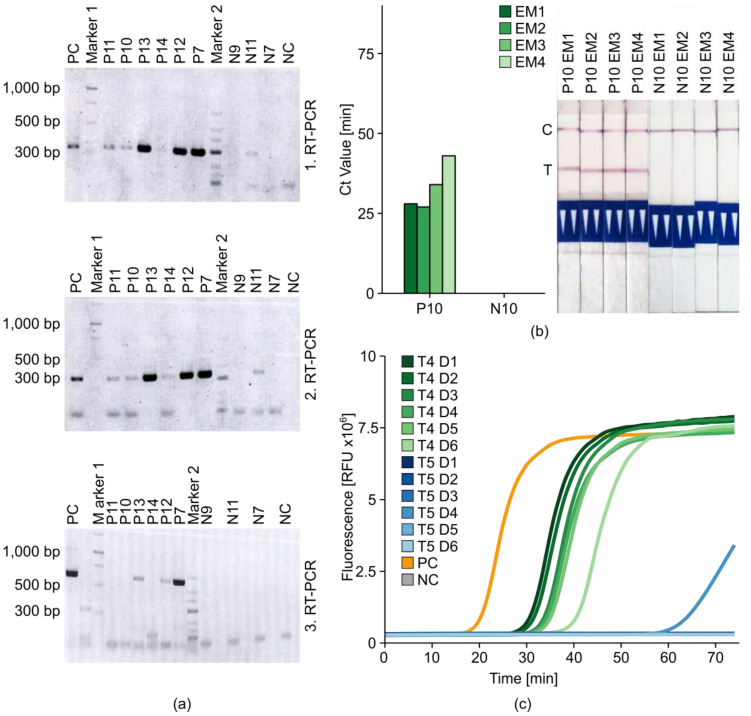
Comparison of simplified RNA extraction methods for amplification by RT-PCR and RT-LAMP. (**a**) Agarose gel of RT-PCR amplification products of purified RNA from incubation samples (InCus) prepared by Extraction Method 4 (EM4) from TRV-positive potato tubers (P11, P10, P13, P14, P12, and P7) and negative tubers (N9, N11, and N7) with primers 1896F + 2181R (1 RT-PCR), 2546F + 2870R (2 RT-PCR), and F3_12092019 + B312092019 (3 RT-PCR). MilliQ water was used as non-template negative control (NC). Marker 1 = GeneRuler 1 kb DNA Ladder, Marker 2 = GeneRuler LowRange DNA Ladder. (**b**) Ct values and lateral-flow dipstick (LFD) analysis of qRT-LAMP assay with FAM/biotin-labeled TRV-PM3 from TRV-positive (P10) and negative (N10) tuber RNA extracted using the standard protocol (Extraction Method 1, EM1), disruption with a hand homogenizer in extraction bags followed by standard RNA purification (Extraction Method 2, EM2), Bioreba extraction (Extraction Method 3, EM3) and column based RNA purification of filtered InCus (Extraction Method 4, EM4). One cycle = 1 min, C = internal dipstick control line, T = TRV positive test line. (**c**) Real-time fluorescence measurement of qRT-LAMP assay of TRV-positive (T4, green) and negative (T5, blue) tuber InCus. Supernatant was diluted 1/100 (D1), 1/200 (D2), 1/400 (D3), 1/800 (D4), 1/1600 (D5) and 1/3200 (D6). DSMZ virus isolate PV-0352 was used as the positive control (PC, orange) and milliQ water as the non-template negative control (NC, gray). RFU = raw fluorescence unit.

Crude wash buffer has proven sufficient in colorimetric LAMP (cLAMP) reactions to detect the fungus *Penicillium oxalicum* present on the surface of grape berries [37]. Therefore, we tested our TRV-LAMP approach using a similar sample preparation method, in which potato tuber and/or leaf tissues are immersed in 50 mL MilliQ water (Extraction Method 4). We incubated nine different tissue samples and used 10 mL of the crude wash buffer, here described as incubation samples (InCus), for RNA purification using the Monarch Total RNA Miniprep Kit (NEB). RNA yields were determined by spectrophotometry, and the samples were then tested using the two-step Takara-RT and iMAXII-PCR protocol with three different primer sets (Figure 8).

The data obtained with two of our three primer pairs were in full agreement with the expected amplicon sizes (Figure 8a). Specifically, primers 1896F + 2181R generated a 286-bp product at 56 °C, and primers 2546F + 2870R generated a 325-bp product at 55 °C. The results were also consistent across laboratories and operators in terms of sensitivity (potential viral loads) for the six infected tubers (P11, P10, P13, P14, P12, and P7) as well as specificity for the three uninfected negative controls (N9, N11, and N7) and the non-template control. Primers F3_12092019 and B3_12092019 used in the third reaction were able to detect three of the six infected samples with no false positives. The nature of the InCu samples was also confirmed using the LFD verification method in comparison to Extraction Methods 1, 2, and 3 (Figure 8b).

Finally, the minimally processed InCus of heavily infected tuber 4 (T4) and healthy tuber 5 (T5) were analyzed directly after passing the crude wash buffer through a 0.2-µm syringe filter but without further purification (Extraction Method 5). The filtered wash buffer was serially diluted from 1/100 to 1/3200 and used as RT-LAMP templates along with TRV inoculum PV-0352 from DSMZ as a positive control and milliQ water as a negative control (Figure 8c). All dilutions of sample T4 were detected correctly and in a clearly concentration-dependent manner (Ct of 1/100 sample was ≈ 25 min; Ct of 1/3200 sample was ≈ 45 min; Ct of the positive control was ≈ 19 min). Only the fourth dilution (1/800) of negative sample T5 was amplified late (T5 D4). We used melt curve and LFD analysis to discriminate between true and false positive results (Table S3). We found that false-positive signals similar to those seen for the T5 D4 dilution sample occurred with higher frequency, strongly depending on the sample quality (e.g., freshness, severity of infection, and storage conditions). Accordingly, the use of InCus for RT-LAMP assays (Extraction Method 5) should be preceded by establishing a range of dilution factors that work well. In our initial experiment, dilutions of 1/200–1/400 performed best in terms of reaction speed (Ct ≈ 26–28 min). More diluted InCus (1/800–1/3200) were still detected, but with a delay of 20 min. For further assay verification, 10 fresh potato samples of unknown TRV-infection status were prepared using Extraction Method 5, and we applied the optimized qRT-LAMP method including 450 mM betaine and HPLC-purified TRV-PM3 (without loop primers), as shown in Table 2.

By combining our minimalized sample-handling method for incubation samples (mini) with the optimized qRT-LAMP method, we categorized 6/10 of the uncharacterized samples as TRV-infected, which is consistent with previous RT-PCR experiments. Passing the InCus through a 0.2-µm syringe filter before the LAMP reactions reduced or abolished the effect of interfering plant-derived components, given that the N11 and N12 negative controls (and healthy tuber samples) did not generate any false positives. Again, the dilution range 1/100–1/400 was ideal for LAMP assays based on InCus (mini-LAMP), especially in terms of the reaction speed and Ct values, detecting all positive samples in 27–40 min for the most concentrated samples or in 33–41 min for the more diluted samples. Our established mini-LAMP-LFD approach and the Bioreba protocol (Extraction Method 3) were able to detect TRV infections precisely and robustly, suggesting the potential for in-field monitoring and disease management.

## 3. Discussion

The detection of potato viruses typically requires field samples to be sent to a well-equipped laboratory for nucleic acid extraction and purification followed by RT-PCR and melt curve analysis. More recently, there has been an effort to replace the complex RT-PCR step with simpler isothermal amplification methods [38,39,40,41,42]. Therefore, we set out to develop a rapid, simple, and inexpensive approach for the detection of potato viruses that (a) minimizes sample processing; (b) provides reliable samples for the rapid and robust on-site diagnosis of infections by isothermal amplification; and (c) allows on-site verification by LFD analysis. Field-grown healthy control and TRV-infected potato tubers were initially used for sample preparation to compare different isothermal amplification techniques. We found that (colorimetric) LAMP was superior to the other methods we tested and selected this approach for the initial development of our TRV-detection assay.

We compared six TRV-LAMP primer sets designed using Primer Explorer to match the TRV-RNA1 region encoding the movement protein. These showed poor performance, so we manually designed primer sets following the guidelines for LAMP primer design by Lucigen. Finding a region suitable for LAMP primer design was challenging due to the natural sequence variation among different TRV isolates. We initially observed that mixed bases (25:25:25:25) at certain sites within the primer sequence improved the sensitivity and performance of the TRV-LAMP assay (TRV-PM1). However, when we replaced these mixed bases with deoxyinosine, which naturally pairs with all four standard DNA bases, this improved the performance of the LAMP assay even further (data not shown). Additional improvements were achieved by replacing the F2 and B2 regions of TRV-PM1 with more conserved target regions (thereby reducing the number of deoxyinosine positions from seven to three), enhancing the specificity of the reaction while maintaining the overall speed (TRV-PM2). This intermediate probe set was further optimized by replacing the B3 component, leading to slightly faster Ct values (≈2 min) and more consistent results on ambiguous samples (TRV-PM3). Parallel work on a PVX-LAMP assay clearly showed that HPLC-purified primer sets and the inclusion of loop primers led to an increase in reaction speed (Table 1). All TRV primer sets were already purified by HPLC (and incorporated deoxyinosine molecules), but the addition of loop primers to TRV-PM3 improved the sensitivity of the assay from an LOD of 78 pg down to 15 pg (Figure 4). Further improvements were achieved by adding betaine to the reaction mix (800 mM for purified RNA, 450 mM for InCus) as well as the UTP/UDG system. Other recommended additives were either neutral or harmful to the reaction, including the addition of high-fidelity DNA polymerase, *Tte* UvrD helicase, and/or DMSO.

Having optimized the LAMP assay, we focused on the development of rapid post-LAMP visualization and verification methods to enable reliable TRV diagnosis in the field. First, we successfully adapted the original LAMP protocol to a colorimetric system, removing the dependence on thermocyclers (measuring fluorescence increments) and/or gel electrophoresis. This allowed the straightforward identification of positive and negative samples based on the presence or absence of color. LAMP methodology is prone to sporadic or non-specific amplification [43,44], especially when using crude plant extracts as a template. Therefore, detection methods must distinguish between specific and nonspecific amplification. To replace the laborious melt curve analysis which is typically used for this purpose, we modified the best-performing primer set for TRV (TRV-PM3 with loop primers) to include FAM/biotin-labeled counterparts compatible with LFD technology (Figure 5). Similarly, we modified the best-performing primer set for PVX (PVX-PM4) with FAM/digoxigenin (DIG)-labeled counterparts (Figure 6). The combination of our LAMP protocol with LFD analysis was successful for both viruses and also facilitated the specific detection of viral pathogens in duplex reactions (Figure 6).

Current protocols for the isothermal detection of potato pathogens rely on the isolation of nucleic acids [45,46,47,48]. Therefore, our final objective was the development of a simple, rapid, and inexpensive sample preparation method that allows RNA isolation in the field without sophisticated equipment. Initially, we compared the standard extraction method based on grinding in liquid nitrogen and processing using the PureLink Plant RNA Reagent (EM1) against the Bioreba potato RNA/DNA extraction set (EM3) and a hybrid method based on the Bioreba extraction bags and homogenizer but the same PureLink reagent used in the standard protocol (EM2). Although the simplified methods performed well, they still rely on commercial reagents. Inspired by work showing that viral RNA recovered from grape berries in a simple washing step is sufficient for colorimetric LAMP assays [37], we iteratively developed a protocol for potato incubation samples (InCus) in which viral RNA is collected from the wash buffer after short incubation (5–15 min). We found that the incubation of tuber slices (TRV) or leaf tissue (PVX) in water was sufficient to release enough high-quality viral RNA for purification and subsequent detection by RT-PCR or RT-LAMP (Figure 8, Table 2). False-positive results for the uninfected sample N11 in the first and second RT-PCRs were most likely caused by contamination, given the negative results of the third RT-PCR (Figure 8a). RNA extracted from InCus by passing through a purification column (EM4) achieved similar sensitivity and specificity to RNA prepared from homogenized tissue (EMs 1–3), and although there was a slight Ct delay, the amplification was still fast enough for detection in less than 50 min (Figure 8b). We also tested InCus without on-column RNA purification (EM5), which was successful within a defined dilution range of 1/100–1/400 (Figure 8c). Finally, we applied the combined protocol of minimal processing (EM5), optimized RT-LAMP, and LFD verification (mini-LAMP-LFD) to randomized positive and negative samples, achieving precise and reliable results (Table 2). To conclude, we demonstrated that our mini-LAMP-LFD method can (1) simplify sample processing to a minimum that does not require any sophisticated laboratory equipment, and (2) generate results that are comparable in terms of precision and reliability to RT-PCR (the gold standard in TRV detection) with a sensitivity of 89% and a specificity of 100%. The estimated cost per sample is 4 € compared to 15 € for the standard RT-PCR assay. This will allow farmers and agricultural workers with limited laboratory training and equipment to monitor their crops in the field and during breeding programs, improving disease management and reducing the economic impact of plant viruses. 

## 4. Materials and Methods 

### 4.1. Potato Tuber Tissue and Controls

TRV-infected and uninfected potato tubers were obtained from field-grown potatoes, and TRV infection was analyzed by RT-PCR. A TRV inoculum (PV-0352) from the DSMZ-German Collection of Microorganisms and Cell Cultures (Braunschweig, Germany) was prepared as a positive control by mixing infected potato leaf tissue with 10 mM Tris (pH 8.0). PVX-infected (PV-0014, PV-0020, PV-0847 and PV-1101) and non-infected (NC-0017) leaf samples were also obtained from the DSMZ and were used for the preparation of extracts by mixing with MilliQ water with subsequent dilution.

### 4.2. Extraction Methods 

The standard RNA extraction method for potato tuber tissue (Extraction Method 1) used PureLink Plant RNA Reagent (Thermo Fisher Scientific, Waltham, MA, USA) according to the manufacturer’s instructions. We also tested two simplified methods. For Extraction Method 2, the grinding of tuber tissue in liquid nitrogen was replaced by the use of extraction bags and a hand-held homogenizer (Bioreba, Reinach, Switzerland). We placed 200 mg of fresh tuber tissue in an extraction bag and added 1 mL of PureLink Plant RNA Reagent. After grinding, 500 µL of homogenate was transferred to a 2-mL tube and RNA was extracted according to the PureLink protocol. For Extraction Method 3, we used the Potato DNA/RNA rapid extraction set (Bioreba, Reinach, Switzerland) according to the manufacturer’s recommendations with minor modifications (the centrifugation step was replaced by a 5-min incubation step at 4 °C for sedimentation). For the preparation of InCus (Extraction Method 4), fresh potato tubers were cut into small pieces and placed in a 50-mL Falcon tube. We added 1 mL of water per 100 mg of tissue and incubated at room temperature for 5–15 min with occasional shaking. The supernatant was passed through a 0.2-µm syringe filter, and the RNA was concentrated on RNA purification columns from the Monarch Total RNA Miniprep Kit (NEB, Ipswich, MA, USA). Filtered supernatant was mixed with an equal volume of Rotisolv HPLC gradient grade ethanol (Carl Roth, Karlsruhe, Germany) before loading onto the columns. Subsequent purification was carried out according to the manufacturer’s recommendations with the omission of one washing step. For the direct analysis of incubation samples, filtered supernatant was diluted 1:100 with water and used directly for amplification (Extraction Method 5).

### 4.3. RT-PCR Amplification Protocol

RT-PCR was carried out using PrimeScript RT Master Mix (Takara Bio, Kusatsu, Japan) according to the manufacturer’s instructions. Each RT reaction comprised 8 µL of template, and 4 µL of cDNA was directly used for subsequent PCR amplification with 2× i-MAX II PCR Master mix (iNtRON Biotechnology, Seoul, Korea) according to the manufacturer’s protocol with the following minor changes: the total reaction volume was reduced to 12 µL and the three primer sets were used at a final concentration of 1 µM. Reaction products were separated by 1% agarose gel electrophoresis together with GeneRuler 1 kb DNA Ladder and GeneRuler Low Range DNA Ladder markers (Thermo Fisher Scientific, Waltham, MA, USA).

### 4.4. Alternative Nucleic Acid Amplification Tests (NAATs)

The alternative NAATs were compared using RNA purified from TRV-infected and uninfected potato tubers. RPA was carried out using the TwistAmp Basic Kit (TwistDx, Maidenhead, UK) or the AmplifyRP Acceler8 Discovery Kit (Agdia, Elkhart, IN, USA). LAMP was carried out using WarmStart LAMP 2× Master Mix (DNA & RNA) (NEB, Ipswich, MA, USA), GspSSD2.0 Isothermal Mastermix (OptiGene, Horsham, UK) or LavaLAMP RNA Component Kit with Dye (LGC, Teddington, UK). The tHDA method was tested using the IsoAmp II Universal tHDA Kit (NEB, Ipswich, MA, USA). For CRISDA, guide RNAs and primers were designed for the TRV movement protein consensus sequence using the Custom Alt-R CRISPR/Cas9 guide RNA design tool (Integrated DNA Technologies, Coralville, IA, USA) and used together with Alt-R S.p. Cas9 D10A nickase v3. CRISDA was carried out as previously described [46] using cDNA prepared from tuber-derived RNA using the PrimeScript RT Master Mix.

### 4.5. Optimization of RT-LAMP Reactions

RT-LAMP reactions were optimized using RNA from TRV-infected and uninfected potato tubers as well as TRV or PVX positive controls from DMSZ. RT-LAMP reactions were prepared with 5 µL WarmStart LAMP 2× Master Mix (DNA and RNA) or WarmStart Colorimetric LAMP 2× Master Mix (DNA & RNA), 0.1 µL 50× LAMP Fluorescent Dye (all from NEB, Ipswich, MA, USA), and 0.5 µL primer mix (2 µM F3/B3 primer, 16 µM FIP/BIP primer, in some cases 8 µM FL/BL primer) in a total volume of 10 µL. RT-LAMP reaction mixtures were incubated at 65 °C for 30–90 min with subsequent melt curve analysis on an ABI7500 Real-Time PCR System (Applied Biosystems, Foster City, CA, USA) or qTOWER 2.0 system (Analytik Jena AG, Jena, Germany).

#### 4.5.1. Primer Design

Multiple alignments of the TRV movement protein (GenBank accessions GQ903771.1, KF758790.1, KJ826365.1, AF166084.1, D00155.1 and AF034622.1) or PVX (45 GenBank sequences) were used to design LAMP primers in Primer Explorer v5 (http://primerexplorer.jp/e/). Initial testing resulted in insufficient and/or nonspecific amplification. Therefore, we manually designed LAMP primer sets using Clone Manager v9 Professional Edition (Sci Ed Software, Westminster, CO, USA). LAMP primer sets were selected according to LAMP primer design guidelines published by Lucigen regarding GC content, Tm values, secondary structure formation (inter-primer and intra-primer homology, dimerization and hairpins), and amplicon length. Primers were ordered from Integrated DNA Technologies (Leuven, Belgium). To reduce nonspecific amplification, initially, TRV-FIPs and BIPs and later all oligonucleotides were purified by HPLC. All LAMP oligonucleotide sequences are listed in Table S1. The target regions of TRV LAMP-PM3 (including loop primer binding sites) are provided in Figure S1.

#### 4.5.2. Additives

To optimize sensitivity and specificity, we tested different concentrations of betaine (0–800 mM) and DMSO (5–10% *v*/*v*), as well as 4 ng/µL *Tte* UvrD helicase (NEB, Ipswich, MA, USA) and 0.012 U/µL Q5 High-Fidelity DNA Polymerase (NEB, Ipswich, MA, USA) per LAMP reaction. As final verification step to reduce the risk of product carryover, we added 700 µM deoxyuridine triphosphate (dUTP, Promega, Madison, WI, USA) and 0.02 U/μL Antarctic thermolabile UDG (NEB, Ipswich, MA, USA). When using dUTP and UDG, RT-LAMP reactions were incubated for 5 min at room temperature prior to amplification at 65 °C.

### 4.6. Lateral-Flow Dipstick (LFD) Analysis

RT-LAMP assays compatible with subsequent LFD analysis included 5′ FAM-labeled FL and 5′ biotin-labeled FIP primers with the same reaction mixture and incubation conditions as for standard RT-LAMP assays. Following each reaction, 3 µL of RT-LAMP products were pipetted onto the sample pads of AMODIA DetectLine Basic dipsticks (AMODIA Bioservice, Braunschweig, Germany) and incubated at room temperature for 5 min. Dipsticks were placed in reaction tubes containing 150 µL of chromatographic buffer and developed for 5–10 min. In the simplified protocol, 150 µL of chromatographic buffer was added directly to tubes containing RT-LAMP amplification products and dipsticks were placed into the samples.

### 4.7. Sensitivity and Applicability of Optimized RT-LAMP

The sensitivity of the first RT-LAMP assay was assessed using RNA from a TRV-positive potato tuber. A two-fold serial dilution was used as the template with 800 mM betaine and primer mix 3. RT-LAMP was performed at 65 °C for 60 min. For the optimized RT-LAMP protocol, the sensitivity was assessed using a 10-fold serial dilution of TRV-positive tuber RNA in triplicate, with 800 mM betaine, the final TRV-PM3 containing loop and labeled primers, dUTP and UDG. RT-LAMP samples were incubated at 25 °C for 5 min and at 65 °C for 60 min with subsequent melt curve analysis.

### 4.8. Duplex RT-LAMP-LFD Assay

For the duplex RT-LAMP with TRV and/or PVX templates, 1 µL of TRV control PV-0352 and/or 1 µL of PVX control PV-0014 (1/4000 dilution) was mixed with biotin/FAM-labeled TRV-PM3 with loop primers (1×) and/or with DIG/FAM-labeled PVX-PM4 with loop primer (0.5×) in a final reaction volume of 10 µL. We also added 40 mM guanidine hydrochloride, UTP, and UDG. Specific amplification was verified by LFD analysis as described above, using AMODIA DetectLine Basic Plus (AMODIA Bioservice, Braunschweig, Germany) for the detection of two amplification products. The lower line represented TRV amplicons (biotin) and the middle line represented PVX amplicons (DIG). 

## Figures and Tables

**Table 1:** Optimization of the potato virus X (PVX) RT-LAMP protocol. We used qRT-LAMP to amplify PVX-positive controls (PV-0014, PV-0020, PV-0847, and PV-1101), negative control (NC-0017), and negative potato tuber RNA (N11 and N12) in two dilutions (1/200 and 1/1600). We used PVX primer mix 4 (PVX-PM4) standard desalted, with HPLC-purified FIP/BIP, or with and additional loop primer. We added 450 mM betaine or 7.5% (*v*/*v*) DMSO to the reaction mix. ^1^ For the Ct value, one cycle = 1 min.

Dilution Factor	Template	Ct Value ^1^ (min)					
		PVX-PM4 (Standard Desalted)		PVX-PM4 (HPLC-Purified)		PVX-PM4 + Loop Primer (HPLC-Purified)	
		450 mM Betaine	7.5% DMSO	450 mM Betaine	7.5% DMSO	450 mM Betaine	7.5% DMSO
1/200	PV-0014	33.79	49.99	28.50	43.29	14.76	33.79
	PV-0020	26.65	51.79	22.70	42.84	12.70	26.65
	PV-0847	21.15	43.69	18.59	36.44	10.65	21.15
	PV-1101	28.85	57.05	27.00	42.52	24.08	28.85
	**mean**	27.61	50.63	24.20	41.30	15.55	27.61
	NC-0017	No Ct					
	N11						
	N12						
1/1600	PV-0014	36.81	55.01	31.11	43.42	16.86	36.81
	PV-0020	26.60	51.07	23.36	43.57	14.88	26.60
	PV-0847	27.47	46.88	24.21	39.61	11.66	27.47
	PV-1101	28.68	25.23	27.43	35.14	20.48	28.68
	**mean**	29.89	44.55	26.53	40.44	15.97	29.89
	NC-0017	No Ct					

**Table 2:** Ct values of TRV RT-LAMP assays on tuber incubation samples (InCus). Filtered InCus prepared using Extraction Method 5 from potato tuber tissue with unknown infection status (T1–10) in two-fold dilutions (1/100–1/3200) were amplified by qRT-LAMP. TRV-negative tubers (N11 and N12) were used as negative controls.

Template	Ct ^1^ Value (min)							
	1/100	1/200	1/400	1/800	1/1600	1/3200	Mean Ct	SD ^2^ Ct
T1	No Ct							
T2	27.43	33.05	35.80	54.73	No Ct	35.30	32.15	n/a ^3^
T3	37.46	40.14	40.63	41.58	50.66	41.98	42.08	4.50
T4	32.64	33.49	35.65	36.14	36.98	42.73	36.27	3.57
T5	No Ct							
T6	34.85	42.43	32.89	No Ct	35.84	No Ct	37.19	n/a ^3^
T7	No Ct							
T8	No Ct							
T9	40.21	40.56	41.05	44.56	53.87	No Ct	44.05	n/a ^3^
T10	34.82	36.23	38.84	41.48	39.16	54.96	40.91	7.27
N11	No Ct	no data						
N12	No Ct							

^1^ Ct: one cycle = 1 min; ^2^ SD: standard deviation; ^3^ *n*/*a*: not available.

## Supplementary Materials

Supplementary materials can be found at https://www.mdpi.com/1422-0067/21/22/8741/s1.

## Abbreviations

InCus	Incubation samples
LAMP	Loop-mediated isothermal amplification
LFD	Lateral-flow dipstick
PVX	Potato virus X
RT-PCR	Reverse transcription polymerase chain reaction
TRV	Tobacco rattle virus
