# Supplementary material for: Fast, Precise, and Reliable Multiplex Detection of Potato Viruses by Loop-Mediated Isothermal Amplification
Source: Int J Mol Sci. 2020 Nov 19;21(22):8741. doi: 10.3390/ijms21228741 (PMC7699554; doi:10.3390/ijms21228741)
Supplement: Supplementary file 1 [file ijms-21-08741-s001.pdf]

International Journal of *Molecular Sciences*

MDPI

Supplementary Data

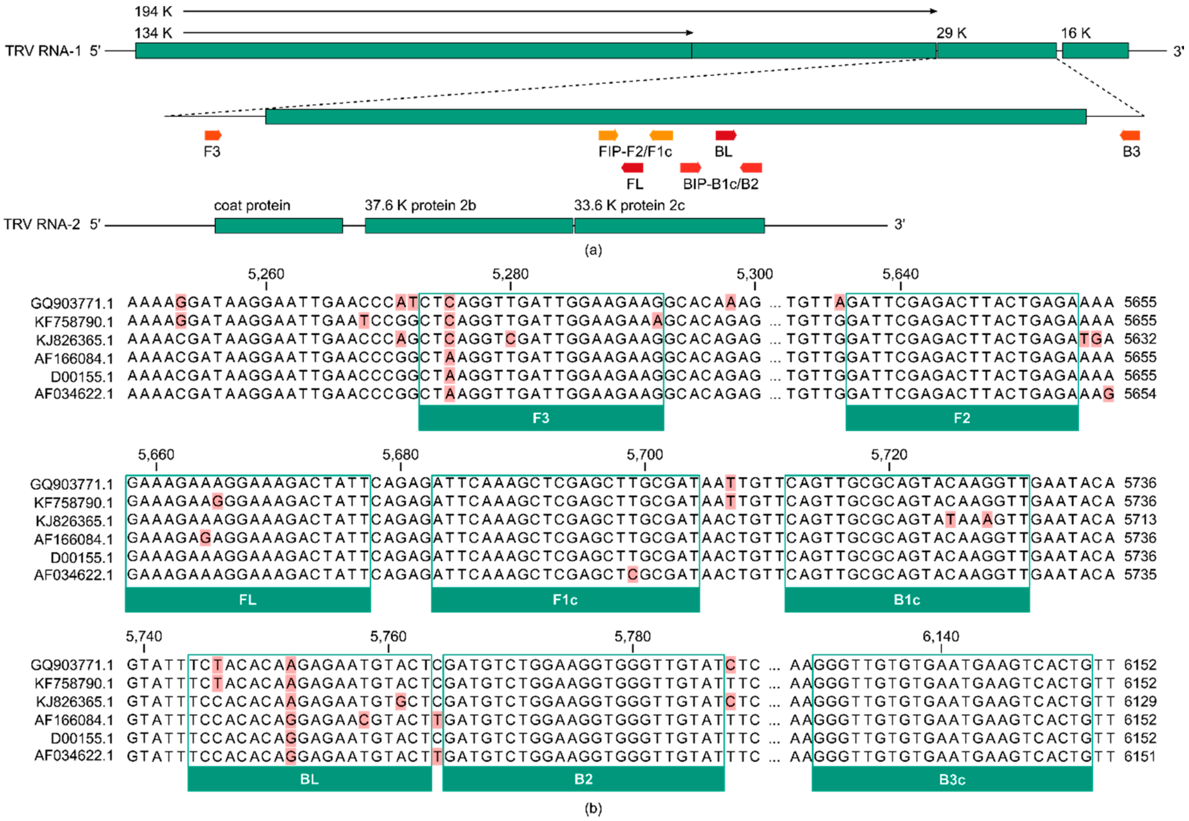

Figure S1. TRV genome and multiple sequence alignment for RT-LAMP primer design. (a) Schematic diagram of the TRV genome with RNA-1 and RNA-2 of TRV isolate MI-1. Open reading frames are depicted as green bars and protein sizes in kDa are abbreviated (K). The position of final TRV RT-LAMP primer set 3 (TRV-PM3) in the 29-kDa movement protein sequence is shown with yellow (FIP), orange (F3, B3), light red (BIP) and dark red (FL, BL) arrows. (b) Multiple alignment of the 29-kDa movement protein sequence (GenBank) showing the exact position of final TRV-PM3.

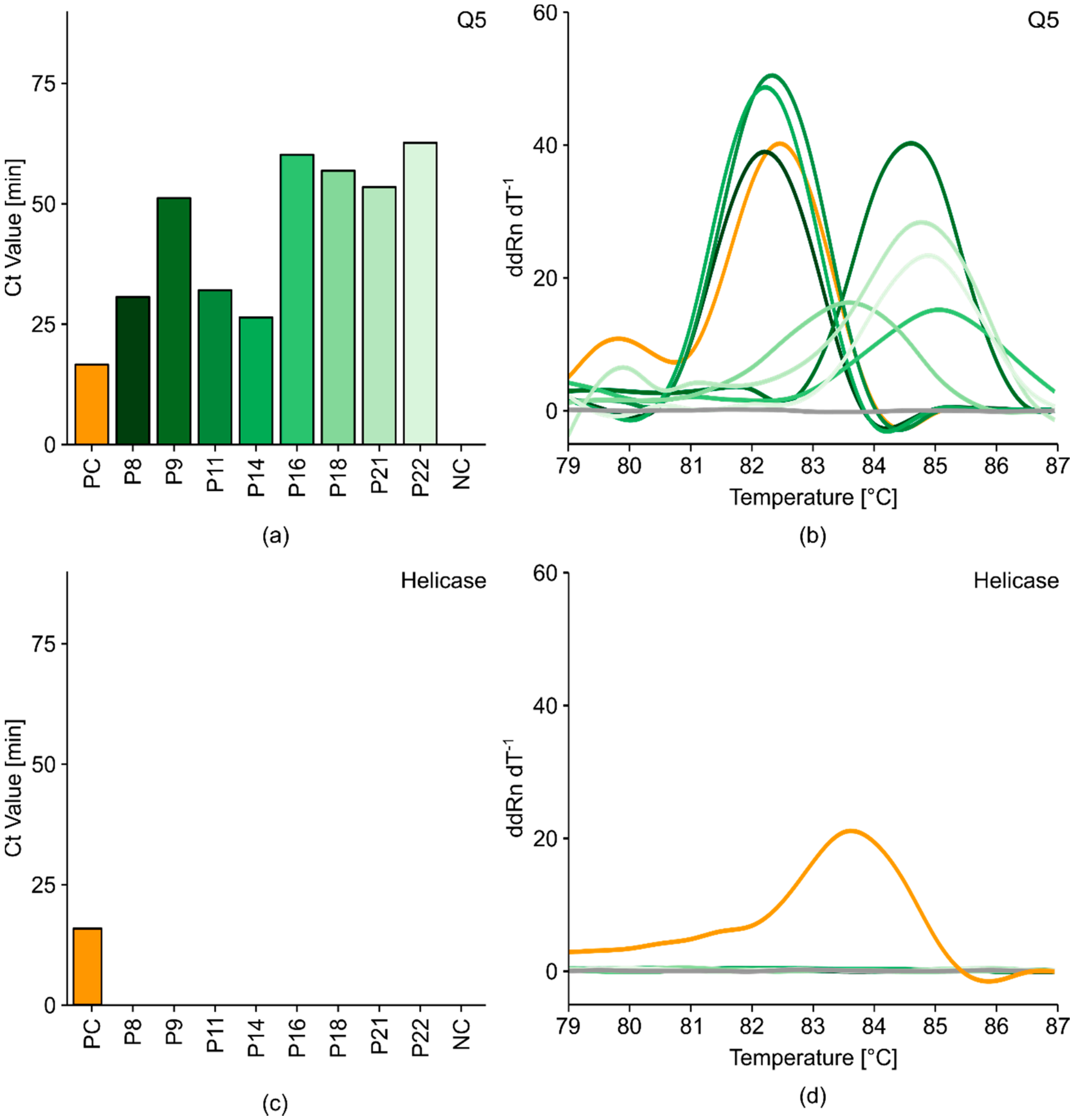

Figure S2. Comparison of Q5 polymerase and Tte UvrD helicase as additives in RT-LAMP assays. RNA from low-copy-number TRV-positive samples was amplified in RT-LAMP reactions with TRV-PM3 plus loop primers using 0.012 U/µL Q5 high-fidelity DNA polymerase (a+b) or 4 ng/µL Tte UvrD helicase (c+d) as additives. DSMZ virus isolate PV-0352 was used as a positive control (PC, orange) and milliQ water as non-template negative control (NC, gray). (a+c) Ct values of RT-LAMP. One cycle = 1 min. (b+d) Melt curve analysis of amplification products (ddRn dT^{-1} = first derivative of normalized fluorescence intensity).

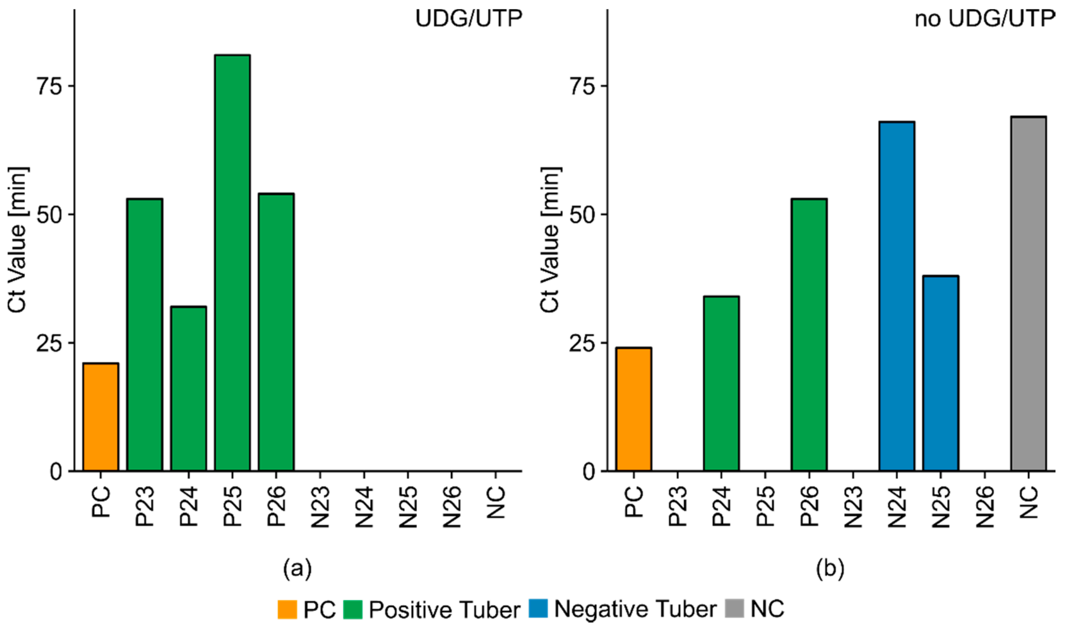

Figure S3. Influence of UDG and UTP on qRT-LAMP assay. Ct values of qRT-LAMP from RNA of TRV-infected (positive, green) and uninfected (negative, blue) tuber tissues with (a) or without (b) UDG and UTP. Positive control (PC, orange) = DSMZ virus isolate TRV PV-0352. Negative non-template control (NC, gray) = milliQ water. One cycle = 1 min.

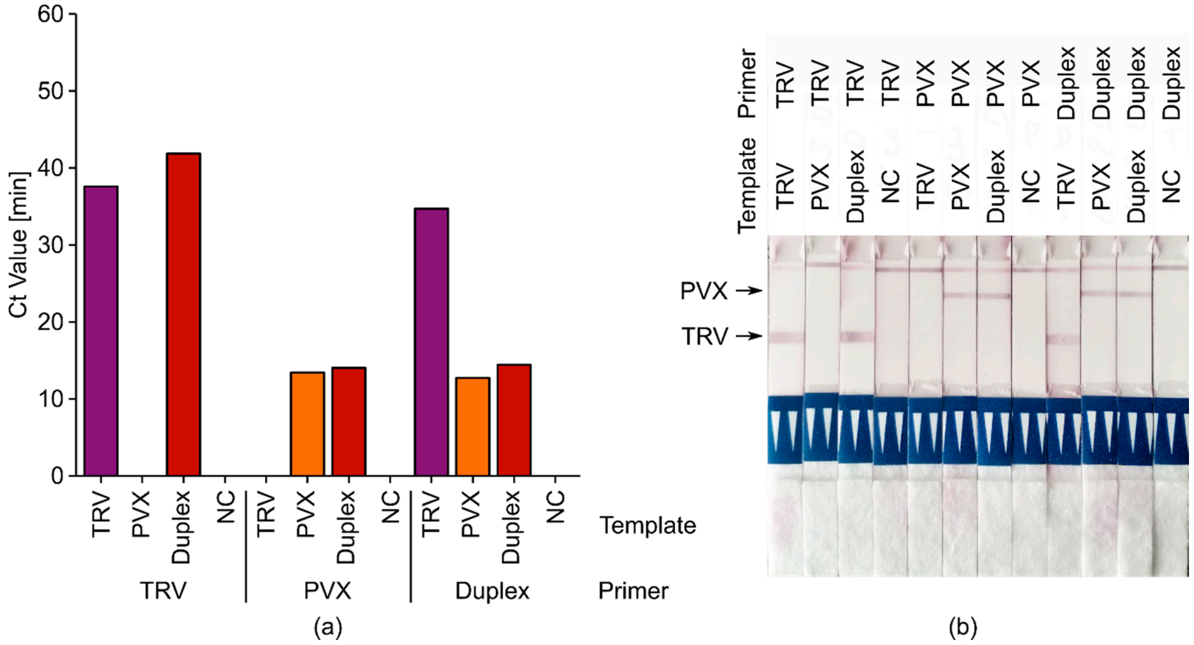

Figure S4. Initial duplex RT-LAMP assay for TRV and PVX. (a) Ct values for the qRT-LAMP analysis of DSMZ virus isolates PV-0352 (TRV, purple), PV-0014 (PVX, orange) or both (duplex, red) with FAM/biotin-labeled TRV-PM3 (TRV), FAM/DIG-labeled PVX-PM4 (PVX) or both (duplex). All primer sets were used at 1× concentration. MilliQ water was used as non-template negative control (NC). One cycle = 1 min. (b) Lateral-flow dipstick analysis of amplification products with two test lines: bottom line = TRV positive, middle line = PVX positive, and upper line = internal dipstick control.

Table S1. Sequences of TRV and PVX primer mixes. N = equimolar mixed bases, dI = deoxyinosine.

Mix	Primer	Sequence
**1 RT-PCR**	1896F	GGGCCCATGTCTTAGACGTTGAGAATCCG
	2181R	ACGTTACTGTCTTCCAACAACAGACCCAGTCC
**2 RT-PCR**	2546F	GCTCAAAATGATGAAATTATGTCTCTGTATCGTG
	2870R	GGGCACTTAAATTGTCCAAGATCAACCTGTTATTGC
**3 RT-PCR**	F3_12092019	GCGATAACTGTTCAGTTGCG
	B3_12092019	CTACGGCCACAACAATTATACAC
**TRV-PM1**	F3	TCTACCTGCNGANACGCC
	B3	GGGTATGTACCGTCACAG
	FIP	ATCGCNAGCTCGAGCTTTGAATGANNTGTTNGATTCGAGACTT
	BIP	CAGTTGCGCAGTANAANGTTGANATACAACCCACCTTCC
**TRV-PM2**	F3	TCTACCTGCNGANACGCC
	B3	GGGTATGTACCGTCACAG
	FIP	ATCGCdIAGCTCGAGCTTTGAATGATTCGAGACTTACTGAGA
	BIP	CAGTTGCGCAGTAdIAAdIGTTATACAACCCACCTTCCAGACATC
**TRV-PM3**	F3	CTMAGGTTGATTGGAAGAAG
	B3	AACCCTTGRGTRCACACG
	FIP	ATCGCdIAGCTCGAGCTTTGAATGATTCGAGACTTACTGAGA
	BIP	CAGTTGCGCAGTAdIAAdIGTTATACAACCCACCTTCCAGACATC
	FL	AATAGTCTTTCCTdIdITCTTTCdIdIdITCTCAGT
	BL	TCdIACACAdIGAGAAdIGTACTdIGATGT
**PVX-PM4**	F3	ACCTTTCTGCTTTCTAGGAAT
	B3	CGTTGAGATATGTATTTACTTCCAT
	FIP	GCTTTGGTGCCGTCTCTGTAGACGTCGGTGACAACATTC
	BIP	GGCAAAACGATGACTGAAATTACAACTCCCCAAATCTAGGGTCA
	FL	CCGTGTGGCAAGCTGTG
	BL	CACAACGGAAAGAACGCAGC

Table S2. Ct and Tm Values of RNA from P11 tuber in different RT-LAMP assays using PM3 and standard RT-LAMP protocol. Tm = melting temperature. [1] Ct: one cycle = 1 min. [2] unspecific melting temperature.

Experiment	Ct[1] Value [min]	Tm Value (°C)
1	No Ct	No Tm
2	38.72	83.25
3	No Ct	No Tm
4	32.06	83.20
5	69.95	84.70 [2]
6	No Ct	No Tm
7	50.34	84.70 [2]
8	34.34	83.20

Table S3. Tm Values and results of LFD analysis of qRT-LAMP assay of TRV-positive (T4) and negative (T5) tuber InCus. PC = positive control DSMZ virus isolate PV-0352, NC = non-template negative control, Tm = melting temperature. [1] unspecific melting temperature.

Sample	Dilution	Tm Value (°C)	LFD Result
T4	1/100	82,92	positive
	1/200	83,11	positive
	1/400	83,11	positive
	1/800	83,30	positive
	1/1600	83,49	positive
	1/3200	83,30	positive
T5	1/100	No Tm	negative
	1/200	No Tm	negative
	1/400	No Tm	negative
	1/800	85,21 [1]	negative
	1/1600	No Tm	negative
	1/3200	No Tm	negative
PC	-	84,06	positive
NC	-	No Tm	negative
